# Molecular Mechanisms Linking High Dose Medroxyprogesterone with HIV-1 Risk

**DOI:** 10.1371/journal.pone.0121135

**Published:** 2015-03-23

**Authors:** Susan C. Irvin, Betsy C. Herold

**Affiliations:** Department of Pediatrics, Albert Einstein College of Medicine, Bronx, NY, 10461, United States of America and Department of Microbiology and Immunology, Albert Einstein College of Medicine, Bronx, NY, 10461, United States of America; Centers for Disease Control and Prevention, UNITED STATES

## Abstract

**Background:**

Epidemiological studies suggest that medroxyprogesterone acetate (MPA) may increase the risk of HIV-1. The current studies were designed to identify potential underlying biological mechanisms.

**Methods:**

Human vaginal epithelial (VK2/E6E7), peripheral blood mononuclear (PBMC), and polarized endometrial (HEC-1-A) cells were treated with a range of concentrations of MPA (0.015-150 μg/ml) and the impact on gene expression, protein secretion, and HIV infection was evaluated.

**Results:**

Treatment of VK2/E6E7 cells with high doses (>15μg/ml] of MPA significantly upregulated proinflammatory cytokines, which resulted in a significant increase in HIV p24 levels secreted by latently infected U1 cells following exposure to culture supernatants harvested from MPA compared to mock-treated cells. MPA also increased syndecan expression by VK2/E6E7 cells and cells treated with 15 μg/ml of MPA bound and transferred more HIV-1 to T cells compared to mock-treated cells. Moreover, MPA treatment of epithelial cells and PBMC significantly decreased cell proliferation resulting in disruption of the epithelial barrier and decreased cytokine responses to phytohaemagglutinin, respectively.

**Conclusion:**

We identified several molecular mechanisms that could contribute to an association between DMPA and HIV including proinflammatory cytokine and chemokine responses that could activate the HIV promoter and recruit immune targets, increased expression of syndecans to facilitate the transfer of virus from epithelial to immune cells and decreased cell proliferation. The latter could impede the ability to maintain an effective epithelial barrier and adversely impact immune cell function. However, these responses were observed primarily following exposure to high (15-150 μg/ml) MPA concentrations. Clinical correlation is needed to determine whether the prolonged MPA exposure associated with contraception activates these mechanisms in vivo.

## Introduction

Injectable hormones such as depot medroxyprogesterone acetate (DMPA), offer high efficacy, convenience, low cost, and privacy for women desiring contraception. DMPA is used by an estimated 35 million women worldwide [1] and is most commonly used in populations where the HIV burden is also greatest (e.g. sub-Saharan Africa and among adolescents). Epidemiological studies suggest that DMPA may increase the risk of acquiring and transmitting HIV and other sexually transmitted infections (STI), although the findings are inconsistent and often represent secondary analyses of data obtained from clinical trials not designed to address the question of DMPA and HIV risk [2–6]. Conducting large-scale clinical trials to examine the impact of hormonal contraceptives on HIV risk is difficult and costly. Defining the potential molecular mechanisms through *in vitro* studies, as presented here, can facilitate the selection of alternative forms of hormonal contraceptive for evaluation by narrowing the field for future clinical trials.

Previously proposed mechanisms that may contribute to increased HIV acquisition include thinning of the epithelium, increased cervical ectopy, alterations in expression of soluble immune mediators either locally or systemically, changes in immune cell populations, and alterations in the vaginal microbiome. However, as recently reviewed [7], data supporting each of these is limited and the results obtained from non-human primate models [8, 9] and clinical studies [10] are inconsistent. For example, marked thinning of the epithelium is observed in macaques treated with a high dose (30 mg) of DMPA [8], whereas studies with doses designed to mimic the clinical setting (3 mg) [9] and human data suggest little or modest effects on epithelial thickness [11–14]. Limited *in vitro* studies, also with conflicting results, have examined the impact of DMPA at the cellular level. However, deleterious effects were observed only with concentrations likely to be supratherapeutic. The precise concentration of MPA that cells or tissue are exposed to following DMPA treatment has not been well defined, although plasma concentrations of 1–7 ng/ml have been reported [15, 16]. An increase in IL-8 and a decrease in RANTES were observed in immortalized ectocervical cells treated with 1 μM (385.5 μg/ml) of MPA combined with 0.02 μg/ml TNF [17]. Higher levels of IL-8 might recruit immune target cells to facilitate infection, whereas lower levels of RANTES, which competes with HIV for binding to CCR5, could reduce mucosal defense. RANTES is also chemotactic for T cells [18], thus lower levels could also be protective by decreasing the number of HIV target cells recruited into mucosal sites of HIV acquisition. HIV infection of peripheral blood mononuclear cells (PMBC) was inhibited by *in vitro* treatment of the cells with 10 μM progesterone and inhibition correlated with reductions in CXCR4 and CCR5 expression on activated T cells [19]. However, in a different study, 1 μM MPA prevented the down-regulation of CCR5 and CXCR4 on T cells after activation *in vitro* and promoted HIV infection of PBMC [20]. In a 12-month longitudinal study of 32 women who initiated DMPA, decreased numbers of CD3+ cells and CD3+ cells expressing CCR5 were observed by immunohistochemistry compared to baseline vaginal biopsies, suggesting that DMPA use may be associated with reduced numbers of HIV target cells in vaginal tissue [21], though the effects of long-term use remain undefined.

Thus, to identify potential mechanisms that might contribute to an increased risk of HIV in the setting of DMPA, we examined the impact of an extensive range of doses of MPA on human vaginal epithelial cells (the first cell encountered by virus during sexual transmission) and PBMC (the primary HIV target cell) in culture models. We also explored whether any observed changes alter HIV infection of immune cells including the Trojan horse-like capture and transfer of HIV from vaginal epithelial to immune cells [22].

## Methods

### Cells and virus

VK2/E6E7 (immortalized vaginal epithelial) and HEC-1-A (human endometrial) cells were obtained from the American Tissue Culture Collection and cultured as previously described [23, 24]. Jurkat-Tat-CCR5 (JT-CCR5) were provided by Q. Sattentau (Sir William Dunn School of Pathology, University of Oxford, Oxford, United Kingdom) and cultured as previously described.[25] U1/HIV-1 (U1) cells, a chronically HIV-1 infected promonocyte cell line, were obtained from the NIH AIDS Research and Reference Reagent Program and cultured as recommended [26]. PBMC were isolated from human leukopacks (New York Blood Center) by gradient centrifugation using Ficoll Paque Plus (Sigma-Aldrich) and were cultured for 3 days (1 x 10^6^ cells/ml) in RPMI 1640 containing 2 mM glutamine, 10% fetal bovine serum (FBS) (HyClone), 100 U of penicillin/ml, 100 U of streptomycin/ml (Gibco), and where indicated, MPA and 5 μg/ml phytohaemagglutinin (PHA) (Sigma-Aldrich). Laboratory-adapted HIV-1_Ba-L_ was grown as previously described [25]. HIV stocks were stored in liquid nitrogen or at -80°C.

### Quantitative reverse transcription PCR (qRT-PCR)

VK2/E6E7 cells were plated in the presence or absence of the indicated concentration of MPA (Greenstone LLC) and were lysed 5 days post-plating, a time point at which there was little impact on cell viability under any of the culture conditions. Total RNA was extracted using the Absolutely RNA Miniprep kit (Agilent). 250 ng of RNA was then reverse transcribed with High Capacity cDNA Reverse Transcription Kit (Invitrogen). RT-PCR was performed using an ABI PRISM 7000 as described [27]. Relative expression was determined by deriving 2^ (-delta delta Ct) relative to the housekeeping gene RPLPO. Commercially available primer and probe sets were purchased from Applied Biosystems. Data is presented as the mean expression + SEM, relative to expression in mock-treated cells (log_10_).

### Detection of cytokines and chemokines

**Fig 1:**
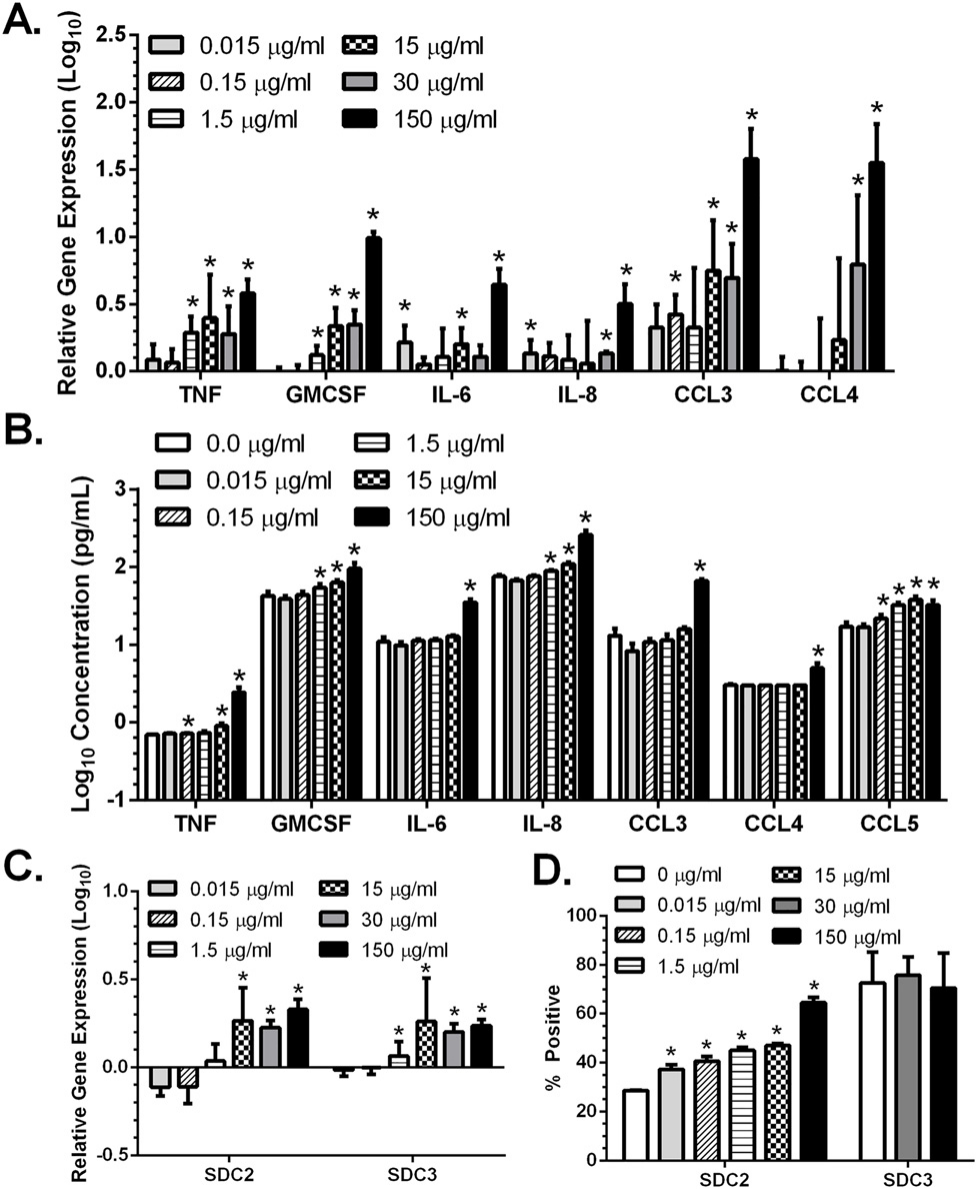
MPA upregulates proinflammatory cytokines and chemokines, and syndecans in vaginal epithelial cells. VK2/E6E7 cells were treated with the indicated concentrations of MPA for 5 days. RNA was extracted, converted to cDNA and analyzed by qRT-PCR for gene expression (A,C) and the culture supernatants were analyzed by Luminex for a panel of cytokines and chemokines (B). The cytokine/chemokine concentrations were log10 transformed to reduce skewness and results are presented as mean + SEM (pg/ml) obtained from two independent experiments. Gene expression was quantified relative to RPLPO and is presented as the mean + SEM relative to mock-treated cells (log_10_) from at least 3 independent experiments (A,C). Alternatively, to examine protein expression, the cells were trypsinized and stained with anti-SDC2-APC or anti-SDC3-APC and analyzed by flow cytometry (D). The results are presented as percentage of APC positive cells after gating on the live cell population and are mean + SEM obtained from two independent experiments (D). Asterisks indicate statistically significantly increased expression compared to mock-treated cells, p<0.05, Student’s t-test.

Cell supernatants were stored at -80°C and plated in duplicate using Milliplex Human Cytokine/Chemokine Panel (EMD Millipore), measured with a Luminex 100 system or MAGPIX system, and analyzed with StarStation 2.3 (Applied Cytometry Systems) or Milliplex Analyst 5.1 (EMD Millipore), respectively, as described previously [28]. For sample concentrations lower than the indicated assay sensitivity, the concentrations were set at the minimum detectable concentration (pg/ml). Data obtained from the two Luminex systems were not combined (Fig. 1B and S1 Fig.) due to differences in assay sensitivities.

### HIV-1 replication in U1 cells

U1 cells were treated with culture supernatants from MPA- or control-treated VK2E6/E7 cells or with 20 U/ml recombinant human IL-6 (R&D Systems), 2.5 U/ml recombinant human GM-CSF (R&D Systems), and 0.5 U/ml or 100 U/ml tumor necrosis factor (TNF) (R&D Systems), alone or in combination. Four days post-treatment, HIV replication was determined by measuring HIV p24 protein by p24 ELISA (NCI-Frederick Cancer Research and Development Center, AIDS Vaccine Program) as previously described [25]. Day 4 was selected as a time point when p24 is readily detected in U1 cells treated with TNF.

### HIV *in trans* infections

VK2/E6E7 cells were plated in the presence or absence of MPA. Five days post-plating, cells were washed and exposed to HIV-1_Ba-L_ (80 ng p24) for 2 hours in the absence or presence of cellulose sulfate (100 μg/ml; Acros Organics), an inhibitor of HIV binding to epithelial cells [29]. Cells were then washed four times to remove unbound virus and drug. JT-CCR5 cells were then added to the same wells and incubated for four hours, transferred to 96-well plates, and incubated for 4 days to allow for viral replication. Supernatants were collected and HIV p24 protein was measured by HIV p24 AlphaLISA assay (PerkinElmer).

### Flow cytometry

Flow cytometry was used to detect cell surface syndecan (SDC) expression and cell proliferation by staining for Ki-67. To evaluate SDC expression, VK2/E6E7 cells were exposed to MPA and then stained (separately) with anti-human syndecan-2-allophycocyanin and anti-human syndecan-3-allophycocyanin (R&D Systems), and then fixed in 4% paraformaldehyde (BD Biosciences). To evaluate cell proliferation, VK2/E6E7 were treated with MPA, stained with LIVE/Dead fixable violet dead cell stain kit (Life Technologies), fixed and permeabilized with Cytofix/Cytoperm Fixation/Permeabilization Solution Kit (BD Biosciences), and finally stained with Alexa Fluor 700 mouse anti-human Ki-67 antibody (BD Pharmigen). To evaluate proliferation of MPA-treated T cells, PBMC were stained with anti-human CD3-phycoethrithrin (PE) (BD Pharmigen), anti-human CD4-FITC (BD Pharmigen), anti-human CD8-APC (BD Pharmigen), and LIVE/Dead fixable violet dead cell stain kit (Life Technologies), cells were then permeabilized and stained with Alexa Fluor 700 mouse anti-human Ki-67 antibody as previously described. Flow cytometric analysis was performed using a BD LSRII (BD Bioscience). FCS files were analyzed using FlowJo version 9.4.9 (TreeStar). Ten thousand live events were acquired per sample; fluorescence minus one negative control for SDC2, SDC3, and Ki-67 were used for gating.

### Polarized culture model

HEC-1-A cells were plated in the apical compartment of Transwell culture plates in the presence or absence of MPA. Transepithelial electrical resistance (TEER) was monitored daily prior to infection. HIV (4.8 ng p24) was added to the apical compartment and JT-CCR5 were added to the basolateral compartment. Alternatively, either JT-CCR5 were infected with HIV (4.8 ng p24) and added to the basolateral compartment or were infected with HIV (4.8 ng p24) for 1 hour at 37°C before being washed and then transferred to the basolateral compartment. Basolateral supernatants were removed on days 1, 3, 5, and 7 post-infection for measuring HIV p24 by HIV p24 ELISA (NCI-Frederick Cancer Research and Development Center, AIDS Vaccine Program) or HIV p24 AlphaLISA (PerkinElmer).

### Statistical analysis

All experiments were conducted in triplicate and at least two independent experiments were performed. GraphPad Prism (version 6; GraphPad Software) was used for statistical analysis of results using Student’s t-test. Differences were considered significant at *P* value <0.05.

## Results

### MPA induced upregulation of proinflammatory cytokines and chemokines and syndecans in vaginal epithelial cells

Vaginal epithelial cells express an array of cytokines and chemokines and contribute to mucosal immunity. To examine the impact of MPA on the immune environment, VK2/E6E7 cells were plated in control media or the indicated concentrations of MPA; at 5 days post-plating, cells were lysed to determine changes in gene expression and parallel supernatants were collected to monitor secreted cytokines and chemokines. Only supratherapeutic doses of MPA (defined as doses such as 15, 30, and 150 μg/ml that substantially exceed the 1–7 ng/ml detected in plasma of women treated with DMPA for contraception [15, 16] consistently and significantly increased gene expression of TNF, GMCSF, IL-6, IL-8, CCL3 (MIP-1α), and CCL4 (MIP-1β) (Fig. 1A). Similarly, significantly more of the same proinflammatory cytokines and chemokines as well as CCL5 (RANTES) were secreted by cells treated with high concentrations of MPA (Fig. 1B and S1 Fig.).

To identify additional mechanisms that could contribute to a link between DMPA and HIV risk, we also examined the impact of MPA on SDC expression. SDCs are heparan sulfate proteoglycans that have been shown to bind and capture HIV and then transfer the virus to immune cell targets in a “Trojan horse” mechanism [30]. Supratherapeutic doses of MPA (15, 30, and 150 μg/ml) significantly increased both SDC2 and SDC3 gene expression (Fig. 1C). Interestingly, even doses of MPA consistent with levels observed following DMPA contraception (0.015 μg/ml) resulted in a significantly greater percentage of SDC2 positive cells (Fig. 1D). The mean fluorescent intensity (MFI) of SDC2 in MPA-treated cells also increased in dose-dependent manner although the increase did not achieve statistical significance (150 μg/ml MPA vs. mock, p-value = 0.08). The fraction of cells positive for SDC3 expression did not increase relative to mock-treated cells (Fig. 1D), however this may reflect higher baseline expression of SDC3 (72.6% SDC3-positive mock-treated cells) compared to SDC2 (28.6% SDC2-positive mock-treated cells).

### Increased cytokine/chemokine response translated to increased HIV replication

**Fig 2:**
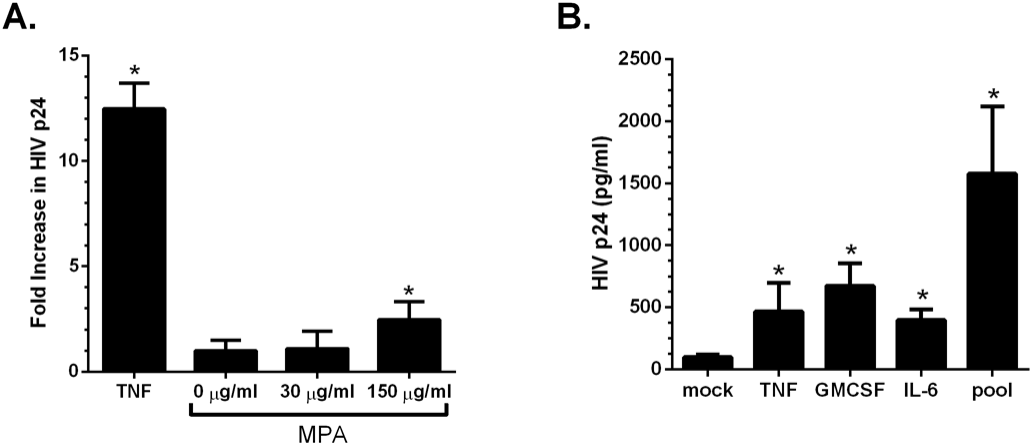
Supernatants from MPA-treated vaginal epithelial cells increase HIV replication in chronically infected U1 cells. VK2/E6E7 cells were plated in the presence or absence of the indicated dose of MPA. At 5 days post-plating, VK2/E6E7 cell supernatants were added to U1 cells and HIV replication was monitored by HIV p24 ELISA on day 4 post-treatment. Controls included supernatants from mock-treated VK2/E6E7 cells (mean HIV p24 1.47 ng/ml) and as a positive control, cells treated with 100 units of recombinant TNF (mean HIV p24 19.365 ng/ml) (A). U1 cells were also treated with recombinant TNF, GMCSF, IL-6, or a pool of these cytokines at concentrations corresponding to levels detected in culture supernatants and HIV replication monitored by measuring p24 levels in supernatants on day 4 (B). Results are mean + SEM of two independent experiments. Asterisks indicate statistically significantly different from mock-treated cells, p<0.05, Student’s t-test.

The chronically HIV-infected monocyte cell line U1 was used to determine the potential effect of the observed increases in cytokines on HIV replication. U1 cells were incubated with culture supernatants from untreated (mock) or MPA treated VK2/E6E7 or, as a positive control, with recombinant TNF; HIV replication was monitored by measuring HIV p24 levels after 4 days. Treatment of U1 cells with supernatants only from high dose (150 μg/ml) MPA-treated VK2/E6E7 cells significantly increased HIV replication (Fig. 2A).

Neutralization or depletion of individual cytokines in culture supernatants is often suboptimal. Therefore, to further understand the relative contribution of individual mediators to the observed increase in HIV replication, U1 cells were exposed to recombinant cytokines at a concentration that corresponded to levels detected in the 150 μg/ml MPA treated VK2/E6E7 cells (Fig. 1A). Compared to U1 cells treated with media alone, TNF, GMCSF, and IL-6 each significantly increased HIV replication and a pool of all three had the greatest impact, suggesting additive effects (Fig. 2B). These cytokines have been previously shown to induce HIV replication [26, 31].

### Transfer of HIV from epithelial to immune cells is increased in association with greater SDC expression

**Fig 3:**
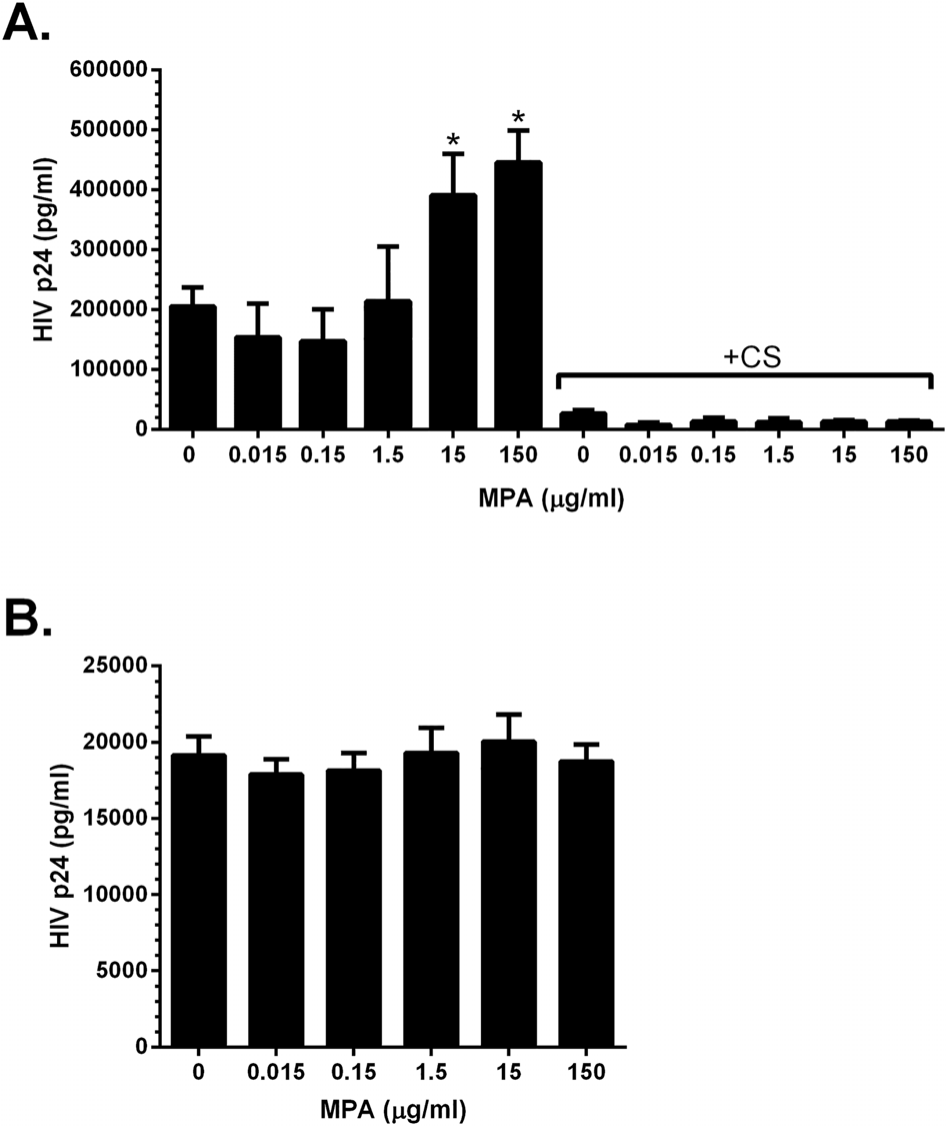
MPA increases HIV *in trans* infection of T cells. VK2/E6E7 cells were plated in the presence or absence of the indicated concentrations of MPA and 5 days post-plating, were exposed to HIV (80 ng p24) in the presence or absence of cellulose sulfate (100 μg/ml). One hour post-HIV exposure, the inoculum was removed, cells washed, and the cells were co-cultured with Jurkat-Tat-CCR5 (JT-CCR5) cells for 4 additional hours to allow virus to be transferred from the VK2/E6E7 to the T cells. The JT-CCR5 cells were then removed and cultured for 4 days and the supernatants analyzed for HIV p24 by ELISA (A). Alternatively, the JT-CCR5 cells were plated in the presence or absence of MPA and then directly infected with HIV (0.8 ng p24) and the supernatants analyzed for HIV p24 on day 4 post-infection (B). Results are mean + SEM of at least two independent experiments; asterisks indicate statistically significant differences relative to mock-treated cells, p<0.05, Student’s t-test.

VK2/E6E7 cells were plated in the presence or absence of MPA for 5 days to allow for maximal SDC expression and then exposed for 1 hour to HIV-1 in the absence or presence of cellulose sulfate, a competitive inhibitor of HIV gp120 binding to heparan sulfate moieties [29]. The cells were then washed and co-cultured with JT-CCR5 T cells and *trans* infection of T cells was monitored by HIV p24 ELISA. There was a significant increase in p24 when T cells were co-cultured with VK2/E6E7 cells treated with supratherapeutic doses of MPA (15 and 150 μg/ml), but no significant difference was observed when VK2/E6E7 cells were cultured with lower MPA doses (Fig. 3A). Transfer of HIV to JT-CCR5 cells was blocked by the addition of cellulose sulfate to the VK2/E6E7 cultures (Fig. 3A). Treatment of VK2/E6E7 cells with high doses of MPA immediately prior to HIV-1 exposure (data not shown) and treatment of JT-CCR5 cells with MPA immediately prior to HIV exposure (Fig. 3B) had no impact on HIV infection. Together these findings suggest that MPA has no direct effect on HIV binding to epithelial cells or infection of T cells, but that the increased SDC2 expression associated with high dose MPA promotes HIV capture and transfer to immune cells.

### MPA decreased cell proliferation

**Fig 4:**
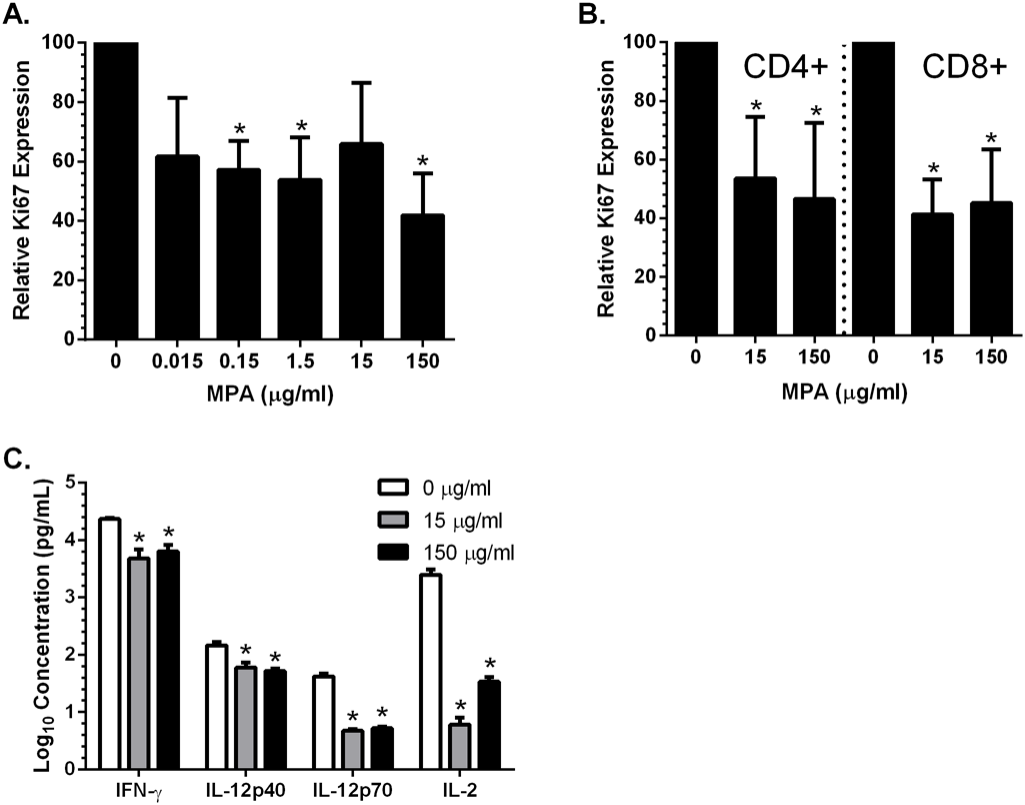
MPA decreases cell proliferation and PBMC response to activation. VK2/E6E7 cells were plated in the presence or absence of the indicated concentrations of MPA and after 5 days in culture, the cells were trypsinized and stained for Ki67 expression by flow cytometry analysis (A). PBMC were isolated and treated with PHA and the indicated concentrations of MPA for 3 days and analyzed by flow cytometry (B). Data is expressed as Ki67+ expression in live epithelial (A) and live CD4+ or CD8+ T cell populations (B) relative to mock-treated cells. Results are mean + SEM of three independent experiments (A,B). PBMC were plated in the presence or absence of MPA. At 3 days post-treatment, PBMC were activated with PHA (5 μg/ml) and incubated for an additional 3 days. Supernatants were collected and analyzed for cytokines and chemokines (C). Results are mean + SEM obtained from two different blood donors (C). Asterisks indicate statistically significantly different from mock-treated cells, p<0.05, Student’s t-test.

Epithelial cell proliferation is an important host defense to maintain the physical barrier against pathogens including HIV. To address the impact of MPA on cell proliferation, VK2/E6E7 cells were exposed to increasing doses of MPA for 5 days and proliferation was monitored by measuring Ki-67 by flow cytometry. There was a significant decrease in Ki-67 in cells treated with doses as low as 0.15 μg/ml MPA (Fig. 4A). Similar findings were observed in both CD4+ and CD8+ T cells when PHA-stimulated PBMC were treated for 3 days with 15 or 150 μg/ml MPA (Fig. 4B).

The decreased proliferative capacity of epithelial cells could facilitate HIV crossing the epithelium to reach submucosal immune cells, whereas the decreased proliferative capacity of activated immune cells might interfere with their immune function. Consistent with this hypothesis, we found that PBMC cultured in 15 and 150 μg/ml MPA and subsequently activated with PHA secreted significantly less cytokines into the culture supernatants including IFN-γ, IL-12p40, IL-12 p70 and IL-2 (Fig. 4C).

### MPA increased HIV transmission across polarized cells

**Fig 5:**
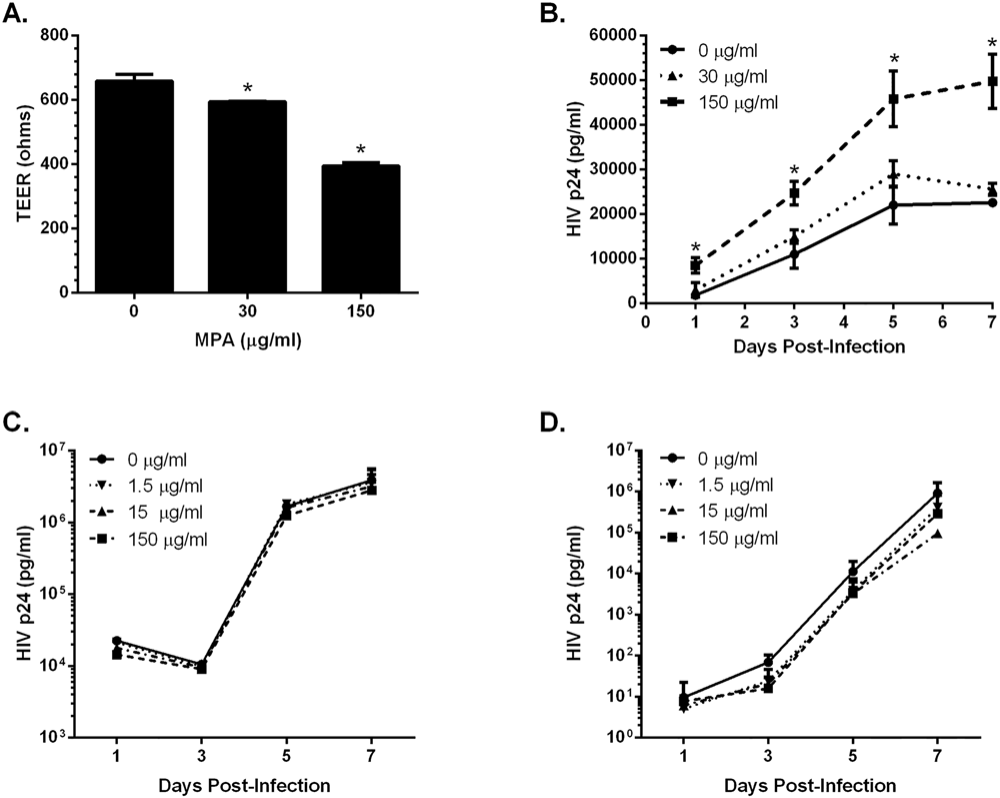
MPA decreased TEER and increased HIV transmission across polarized cells. HEC-1-A cells were plated in the apical chamber of transwell culture plates in the presence or absence of the indicated concentrations of MPA and TEER was monitored daily until at least 400 ohms was maintained. Representative TEER results from duplicate wells on Day 4 are shown (A). HIV (40 ng p24) was added to the apical chamber and JT-CCR5 cells were added to the basolateral chamber and transmigration and infection were monitored by analyzing basolateral culture supernatants for p24 by ELISA on days 1, 3, 5, and 7 post-addition of HIV to the apical compartment (B). Alternatively, JT-CCR5 cells were exposed to HIV (4.8 ng p24) either when cells were added to the basolateral chamber (C) or were exposed to HIV in a 96-well plate, washed, and then added to the basolateral chamber (D) and HIV replication was again monitored over time. Asterisks indicate statistically significant differences compared to mock-treated cells, p<0.05, Student’s t-test.

We evaluated the impact of MPA on the ability of HIV to cross from the apical (AP) to the basolateral (BL) compartment in a Transwell culture model with HEC-1-A cells. This model has been previously used to address toxicity of topical microbicides.[32] After 4 days in culture, mock-treated HEC-1-A cells achieved a TEER of 659 ± 14 ohms, whereas there was a dose-dependent lower TEER achieved in MPA treated cells (Fig. 5A). The reduction in TEER in cells treated only with high dose MPA (150 μg/ml) was associated with an increase in HIV-1 migration when virus was added to the AP compartment and the ability of virus to infect JT-CCR5 cells cultured in the BL compartment was assayed (Fig. 5B). To determine if the increased HIV p24 measured in BL compartment reflected an increase in HIV transit from AP to BL side or an indirect effect of soluble factors released by the HEC-1-A cells in response to MPA that might promote HIV infection or replication, the assay was modified. Rather than adding HIV to the AP as described above, once TEER of at least 400 ohms was achieved, JT-CCR5 cells were added to the BL and directly infected with HIV-1 (Fig. 5C) or JT-CCR5 cells were infected with HIV-1 in a separate plate, washed, and then added to the BL compartment (Fig. 5D). There was no significant difference in HIV replication in JT-CCR5 cells added to the BL chamber of MPA treated or untreated HEC-1A cultures. Taken together, the results suggest that the increase in p24 levels observed when HIV-1 was added to the AP chamber of high dose MPA-treated HEC-1A cells reflects an increase in HIV transit from AP to BL compartment, which likely reflects the lower TEER obtained in the setting of MPA. The inability of MPA-treated HEC-1A to achieve a high TEER and efficiently function as a barrier to HIV-1 may reflect reduced cell proliferation as noted in Fig. 4 or altered cell-cell junctional proteins. However, no differences in expression of cell-cell junctional genes (claudin-1, claudin-5, demoglein-1, desmoglein-2, occludin, zona occludens 1, and zona occludens 2) were observed in MPA-treated HEC-1-A cells (data not shown).

## Discussion

One of the potential risk factors for HIV acquisition and transmission is the use of DMPA, although epidemiological findings are inconsistent. However, few studies have explored the potential biological mechanisms and this knowledge may facilitate the selection and evaluation of alternative forms of hormonal contraception. The current study identified three potential mechanisms that may link DMPA with HIV risk, although the effects were primarily observed in response to brief exposure to high doses of MPA that likely exceed the concentrations achieved following contraceptive administration. Thus, clinical studies are needed to determine if the mechanisms identified *in vitro* are recapitulated when there is prolonged exposure to therapeutic doses of DMPA or other forms of hormonal contraception.

First, culture of epithelial cells with high doses of MPA for 5 days increased RNA and protein levels of several inflammatory cytokines and chemokines. We elected to use this more extended culture time to simulate what might happen in the setting of sustained exposure. Importantly, treatment of cells with doses as high as 150 μg/ml of MPA for 5 days was not cytotoxic and did not increase apoptosis (data not shown). The increased levels of proinflammatory cytokines/chemokines were sufficient to activate HIV replication in U1 cells. However, this response was only observed when U1 cells were incubated with culture supernatants harvested from epithelial cells treated with doses of MPA that far exceed the plasma concentrations of 1–7 ng/ml observed in women treated with DMPA.[15, 16] The observation that the increase in inflammatory molecules is only observed with what are presumed to be supratherapeutic dosing is consistent with results of a recent prospective study showing no increase in plasma or cervical HIV RNA levels in association with DMPA [33].

Second, exposure to MPA increased the expression of SDC resulting in increased transfer of HIV from epithelial to T cells. Increased SDC protein was detected by flow cytometry even in response to doses of MPA that may occur after DMPA injection, although gene expression was upregulated only by high doses. Heparan sulfate (HS) side chains of SDC capture HIV particles and transfer the virus to T cells through a Trojan horse-like mechanism [22, 30, 34, 35]. The interaction between HIV gp120 and HS stabilizes the HIV virion for at least 5 days, creating a window of opportunity for HIV to infect lymphocytes recruited into or migrating through the FGT [22, 30]. Notably, in ongoing studies we observed increased SDC2 (p<0.001) and SDC3 (p = 0.056) gene expression in genital tract tissue from C57BL/6 mice after DMPA treatment (unpublished). Increased SDC expression was also observed in a microarray analysis of vaginal epithelial tissue isolated from DMPA-treated macaques compared to macaques in the follicular phase (SA Vishwanathan and EN Kersh, personal communication, December 4, 2013). Studies with human tissue are needed to determine if these findings translate to the clinical setting. An increase in SDC expression could also facilitate infection by other STI including herpes simplex virus (HSV), *Neisseria gonorrhoeae*, and *Chlamydia trachomatis* as each of these pathogens binds to HS moieties on SDC [36, 37]. Moreover, HSV upregulates SDC expression [38] and thus could act synergistically with DMPA to facilitate HIV acquisition.

Third, we found that MPA treatment was associated with a significant decrease in Ki-67 expression, a cell proliferation marker. This may translate to an inability to maintain an effective barrier, as evidenced by the transwell model, or to repair abrasions, which might occur during sexual intercourse or from epithelial lesions. The decrease in cellular proliferation was also observed *in vitro* with PBMC, which could adversely affect immune cell function as evidenced by the decreased cytokine response to PHA activation in MPA-treated PBMC. The finding of decreased Ki-67 differs from a prior study, which found an increase in the number of Ki-67+ epithelial cells in genital tract biopsies obtained from women 12 weeks after a single 150 mg IM dose of DMPA compared to biopsies obtained during the follicular or luteal phase of the menstrual cycle [12]. The increase at the 12 week time point, may reflect a response to impaired proliferation during the peak of MPA exposure. Clinical studies with biopsy sampling at different times following DMPA dosing are needed to test this notion. The decrease in Ki-67 observed here is consistent with other studies suggesting impaired immune function. For example, CD3+ T cells and CD14+ monocytes secreted decreased levels of IFN-γ and TNF, respectively when the cells were pretreated with MPA prior to activation [20]. Similarly, another study found decreased MIP-1α, MIP-1β, and RANTES secretion from CD8+, but not CD4+ T cells, treated with MPA and simultaneously activated with PHA [19].

The extent to which each of the potential mechanisms identified in this and other studies translate to the clinical setting requires further studies. While a decrease in Ki67 and increase in SDC protein were observed following exposure to 15 ng/ml of MPA, higher doses of MPA were required to trigger an increase in HIV replication in U1 cells or to increase transfer of HIV from epithelial to T cells. These high doses greatly exceed the previously reported peak plasma concentrations of MPA of 1 to 7 ng/ml following DMPA treatment [15, 16]. Other *in vitro* studies have used even higher doses (e.g. 385.5 μg/ml) [1, 17], resulting in data that may not be applicable to the in vivo effects of DMPA. However, the cumulative effects of sustained MPA exposure coupled with the possible additive or synergistic effects of other STI on cytokines, chemokines, SDC and epithelial barrier function may act in concert to increase HIV risk, though the degree of synergism remains to be determined by future in vivo studies. Another limitation of the study is that the cells were treated with MPA only, not with a combination of MPA and low level estradiol, which may better recapitulate the clinical situation of low levels (40 pg/ml) of estradiol in the plasma of women on DMPA contraception [15].

Further research is also needed to test whether alternative forms of hormonal contraception have similar *in vitro* effects and whether these effects are mediated by interactions with progesterone or glucocorticoid receptors. This is particularly important because MPA, but not, for example, levonorgestrel (LNG), binds to the glucocorticoid receptor, which is expressed by many types of immune cell types [39] and engagement of the glucocorticoid receptors may interfere with innate and adaptive immune responses [40]. Defining the in vitro response to other candidate hormonal contraceptives could inform the design of large-scale clinical trials in development comparing different forms of progesterin as contraceptives in women at high risk for HIV. For example, there is little data on the impact of norethisterone oenanthate (NET-EN) or LNG on mucosal or systemic immunity. LNG is another progestin-based contraception currently available for women formulated as an intrauterine device (Mirena), which offers sustained contraception, is safe, efficacious, and highly accepted in young women. However, there are no data examining its impact on HIV risk. Combination intravaginal rings designed to deliver antiretroviral drugs for HIV pre-exposure prophylaxis with LNG for contraception are in development. It may be prudent to evaluate these alternative hormonal contraceptives and multipurpose combination products in culture systems and animal models prior to embarking on further clinical development.

## Supporting Information

S1 Fig MPA upregulates proinflammatory cytokines and chemokines in vaginal epithelial cells. VK2/E6E7 cells were treated with the indicated concentrations of MPA for 5 days and the culture supernatants were analyzed by Luminex for a panel of cytokines and chemokines. The cytokine/chemokine concentrations were log10 transformed to reduce skewness and results are presented as mean + SEM (pg/ml) obtained from three independent experiments. Asterisks indicate statistically significantly different compared to mock-treated cells, p<0.05, Student’s t-test.
(TIF)
